# Clonal selection versus clonal cooperation: the integrated perception of immune objects

**DOI:** 10.12688/f1000research.9386.1

**Published:** 2016-09-05

**Authors:** Serge Nataf

**Affiliations:** 1 Bank of Tissues and Cells, Lyon University Hospital (Hospices Civils de Lyon), CarMeN Laboratory, INSERM 1060, INRA 1397, INSA Lyon, Université Claude Bernard Lyon-1, University Lyon-1, Lyon, France

**Keywords:** theoretical immunology, neuroimmunology, sensory perception, immunity, brain, T-cells

## Abstract

Analogies between the immune and nervous systems were first envisioned by the immunologist Niels Jerne who introduced the concepts of antigen "recognition" and immune "memory". However, since then, it appears that only the cognitive immunology paradigm proposed by Irun Cohen, attempted to further theorize the immune system functions through the prism of neurosciences. The present paper is aimed at revisiting this analogy-based reasoning. In particular, a parallel is drawn between the brain pathways of visual perception and the processes allowing the global perception of an "immune object". Thus, in the visual system, distinct features of a visual object (shape, color, motion) are perceived separately by distinct neuronal populations during a primary perception task. The output signals generated during this first step instruct then an integrated perception task performed by other neuronal networks. Such a higher order perception step is by essence a cooperative task that is mandatory for the global perception of visual objects. Based on a re-interpretation of recent experimental data, it is suggested that similar general principles drive the integrated perception of immune objects in secondary lymphoid organs (SLOs). In this scheme, the four main categories of signals characterizing an immune object (antigenic, contextual, temporal and localization signals) are first perceived separately by distinct networks of immunocompetent cells.  Then, in a multitude of SLO niches, the output signals generated during this primary perception step are integrated by TH-cells at the single cell level. This process eventually generates a multitude of T-cell and B-cell clones that perform, at the scale of SLOs, an integrated perception of immune objects. Overall, this new framework proposes that integrated immune perception and, consequently, integrated immune responses, rely essentially on clonal cooperation rather than clonal selection.

## Introduction

Evolution has endowed the human species with the most sophisticated immune and nervous systems. Maintenance of our internal homeostasis and adaptation to our external environment rely essentially on the ability of both systems to sense, memorize and react to a large variety of input signals. These crucial functions are supported by a common organizational grounding base consisting in complex networks of specialized cells that communicate in specific anatomical sites. Similarities between the immune and nervous systems were first highlighted by the immunologist Niels Jerne who introduced the terms “recognition”, “memory” and “learning” in the immunological vocabulary ^1, 2^. However, since then, such an analogy-based reasoning was mostly used to demonstrate reminiscent molecular mechanisms between immune and neuronal synapses ^3, 4^. Only few works attempted to further theorize the immune system functions through the prism of neurosciences. In this framework, the cognitive immunology paradigm proposed by Irun Cohen ^5, 6^ is undeniably a key contribution that notably led to the concept of physiological auto-immunity ^7, 8^. In particular, Irun Cohen proposed that naturally occurring auto-antibodies provide an indispensable immune system's representation of our body, the immunological *homunculus* ^5^, which resembles its neural counterparts, the somatosensory *homunculus*. Thereafter, other works similarly apprehended immunity as a cognitive process and brought about the emergence of computational immunology ^9, 10^. Nevertheless, the line of thought initiated by Jerne appears not to have been nourished by the major conceptual and experimental advances that cognitive neurosciences provided in the last two decades. This context offers a unique opportunity to revisit and explore analogies between the nervous and immune systems in the light of such discoveries. The recently formulated concept of a sensory immune system ^11^ falls into this re-thinking strategy.

### Cooperative neuronal networks support the integrated perception of visual objects

**Figure 1.**
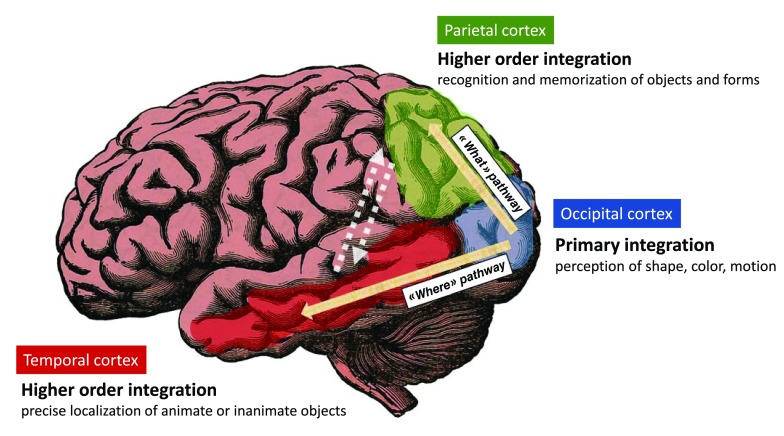
Brain visual pathways. The signals related to the shape, color and motion of visual objects are integrated by specialized brain neuronal populations residing in the primary visual cortex i.e the superficial neuronal layers of the brain occipital lobe. Output signals are then generated that instruct other cortical areas for higher order integration tasks. The “What” pathway connects the primary visual cortex to areas of the temporal cortex that are essential to the recognition and memorization of objects and forms. The “Where” pathway connects the primary visual cortex to areas of the parietal cortex that support perception of precise localization. The interconnections between higher-order visual areas (dashed arrows) as well as other brain areas not highlighted here, allows a fully-integrated perception that takes into account the “What”, “Where”, “How” and “When” features of a visual object.

Developing further the concept of “perceptive immunity” requires beforehand to provide a basic description of the main mechanisms allowing our brain to perceive sensory inputs. Let us choose the example of visual perception. When considering the perception of a given visual object, different categories of input signals that relate with the shape, color, and motion of this object are captured and integrated independently by distinct neuronal populations. These specialized neuronal networks reside in the so-called primary visual cortex, in the superficial neuronal layers of the brain occipital lobe) ^12, 13^. Importantly, such a **primary perception** induces the generation of output primary signals (electro-chemical by nature) that converge toward neurons localized in the so-called visual association areas also named higher-order visual areas ^14, 15^ ( Figure 1). There, these specialized neuronal populations capture and integrate varied combinations of output primary signals to perform an **integrated perception** of visual objects. Two major pathways allowing the convergence of output primary signals toward higher-order areas are well characterized: i) the “What” pathway targets associative areas in the temporal cortex and is essential to the recognition and memorization of visual objects ^14^, ii) the “Where” pathway targets associative areas of the parietal cortex and supports the perception of precise localization ^14^. Eventually, the interconnections between high-order visual areas allows a fully-integrated perception that takes into account the nature, localization, context and time-related features of a visual object ^14^.

Thus, visual perception requires not only a specialization of cells depending on their ability to perform primary vs integrated perception tasks but also tight cooperation between neuronal networks. **Primary perception** allows distinct features of a visual object (shape, color, motion) to be perceived separately ^12, 13^. **Integrated perception** allows a visual object to be perceived as a whole via the integration of distinct categories of primary signals ^14, 15^.

### Non-neuronal cells are also essential to visual perception

It also important to underscore that the operability of any neuronal network, would it be involved or not in sensory perception, depends on non-neuronal cells that locate in close vicinity to neurons. Astrocytes exert a tight control of interneuronal synaptic transmission ^16, 17^ and microglia, the resident macrophages of the brain, proceed to a selective trimming of functionally irrelevant or supernumerary synapses ^18, 19^. In addition, the blood flow in small arteries and capillaries of the brain is exquisitely tuned by a mechanism of neurovascular coupling that finely adjusts the supply of blood-derived oxygen and glucose to the needs of neuronal networks ^20, 21^.

### The immune system perceives more than antigens and danger signals: a proposed definition of immune objects

Postulating the existence of analogies between the immune and visual systems implies first that the counterpart of visual objects are immune objects. If so, immune objects cannot be simply reduced to an antigen +/- danger signals. Indeed, in accordance with the principles of immunogenicity previously enunciated by Rolf Zinkernagel ^22^, one may propose that an immune object (IO) is defined by the association of at least 4 categories of signals: antigenic, contextual, temporal and localization signals. Establishing a parallel between visual and immune perception also implies that the perception of an IO relies first on **primary perception** tasks followed by an **integrated perception** step. It is proposed that distinct IO-related features (antigen, context, localization, time-related signals) are perceived separately by distinct networks of immunocompetent cells in a myriad of SLO niches. Then, output signals generated by such a primary perception step converge toward T-cells which, at the scale of SLOs, perform and orchestrate the integrated perception of IOs.

### The primary perception of immune objects is a cooperative task operated by a large range of immunocompetent cells

**Figure 2.**
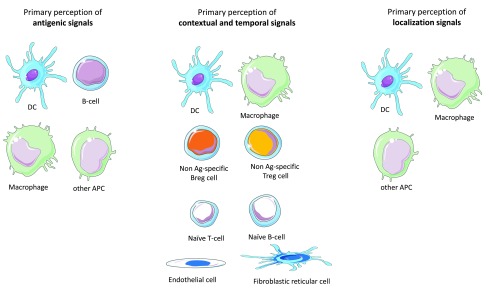
Primary perception of antigenic, contextual, localization, and temporal signals. The antigenic, temporo-contextual and localization signals that characterize an immune object are perceived separately by specific populations of cells that perform a primary perception task. The output signals generated by these cells will then instruct an integrated perception step essentially performed by TH-cells. The primary perception of antigenic signals is performed by DCs, macrophages, B-cells and any APC that may reside or migrate in lymph nodes. The primary perception of contextual and temporal signals including notably DAMP, PAMP and cytokines are performed by a large range of immune cells or non-immune cells that reside in SLOs. In addition, immune cells that target SLOs in a context- and time-dependent fashion also perform a primary perception of temporo-contextual signals. These cells include notably Treg and Breg cells irrespective of their target antigens as well as, to some extent, naive B- or T-cells. Finally, the primary perception of localization signals is essentially performed by APCs that derive from the immune object microenvironment.

As previously proposed ^11^, SLOs are likely to be the main anatomical sites where the primary and integrated perception of IOs take place. Below is an attempt to categorize immunocompetent cells according to their functions in the **primary perception of IOs** and the ensuing generation of primary output signals ( Figure 2).

1) The primary perception of antigenic signals is essentially performed by antigen-presenting cells (APCs) would they belong or not to the dendritic cells (DC) lineage ^23^. While tissue-resident DCs are the first line cells exerting such a function, a flurry of APCs that reside or migrate in SLOs also participate to the primary perception of antigens. Depending on the intrinsic properties harbored by APCs with regard to antigen processing and co-stimulation, the primary perception of antigens will result in the presentation of distinct epitopes and the expression of varied combinations of accessory molecules ^23^.
2) Contextual signals are highly diverse in nature and may combine in many different ways under physiological or pathological conditions (development, ageing, trauma, degeneration, infection, cancer…etc.). Danger-associated molecular patterns (DAMP), pathogen-associated molecular patterns (PAMP) and cytokines, which form the great majority of contextual danger signals, bind receptors harboring a large expression pattern in SLOs. The primary perception of contextual signals is thus likely to involve not only immune cells but also SLO-residing endothelial cells and stromal cells ^24– 26^. Output primary signals consist in a larger array of cell surface and soluble factors that instruct the integrated perception step. Moreover, a variety of immune cells that target SLOs in a context-dependent fashion participate to the primary perception of contextual signals and provide part of the primary response to such signals. These include notably Treg and Breg cells irrespective of their antigen specificity ^27, 28^, NK cells ^29^, polymorphonuclear cells ^30^, monocytes ^31, 32^, innate lymphoid cells ^33, 34^ as well as naive T or B lymphocytes ^35, 36^. Overall, the primary perception of contextual signals is a cooperative task performed by a large array of cell types that reside in SLOs or migrate toward SLOs. These cells generate of whole of soluble or membranous output signals that instruct the integrated perception of IOs.
3) The primary perception of localization signals is essentially performed by dendritic cells and macrophages that are drained from the IO's tissue environment ^37– 40^. The output signals generated by these tissue-derived APCs will imprint the homing properties of T-cells ^38, 39, 41^ and orientate in a tissue-specific manner the polarization of TH cells (notably toward TFH cells) ^42^. Interestingly, recent findings indicate that stromal cells also play a major role in the primary perception of localization signals ^43, 44^.
4) Temporal cues are provided by the duration of the antigenic, contextual and/or localization signals. Sudden vs chronically-installed IOs are not equally seen by the immune system according to the discontinuity theory ^45^. However, one may also consider that the distinct patterns of tissue-derived cytokines characterizing acute vs subacute vs chronic inflammation provide crucial time-related inputs. In any case, as proposed above for contextual signals, the primary perception of temporal signals is a cooperative task performed by a large array of cell types in SLOs.

### The integrated perception of immune objects is essentially performed by TH-cells

The highly complex spatial organization of SLOs, essentially determined by stromal cells ^24, 46– 48^, is currently viewed as a means to tightly control the movements of cells and fluids in SLOs ^49, 50^. Such a stromal scaffold formed by endothelial cells and fibroblastic reticular cells also provides a histologic support to a number of niches that exhibit distinct microenvironments. For a given IO, there is indeed a myriad of APCs (subsets of DCs, macrophages, B-cells, other APCs) that interact with naïve or central memory T-cells in specific niches localized, for lymph nodes, in the paracortical ^51^, subcapsular ^52, 53^ or medullar zone ^54, 55^. Such niches are formed by partially overlapping yet distinct compositions of immunocompetent cells that proceed to the primary perception of antigenic, contextual, localization and temporal signals. It can be proposed that in each of these niches, immunocompetent cells having proceeded to the primary perception of an IO generates output signals that converge toward T-cells bearing cognate TCRs. By this mean, T-cells (TH cells or T cytotoxic CD8 T-cells activated via cross-presentation) capture and integrate at the single cell level a whole of signals that relate with the antigenic, contextual, localization and temporal features of an IO.

### The integrated perception of immune objects relies on clonal cooperation rather than clonal selection

**Figure 3.**
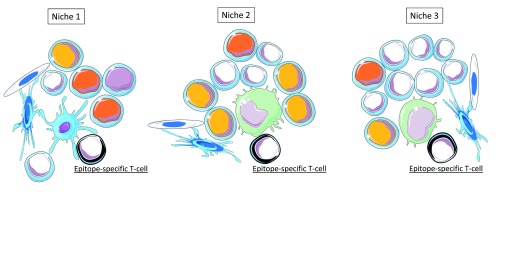
Distinct SLO niches provide distinct combinations of output signals resulting from primary perception tasks. Epitopes presented in the context of MHC molecules are the output signals resulting from the primary perception of antigens. In SLOs, multiple epitopes derived from an immune object are recognized by multiple epitope-specific T cells in distinct niches. These niches are formed by stromal cells (endothelial cells and fibroblastic reticular cells) and by partially overlapping combinations of immune cells performing the primary perception antigens, temporo-contextual signals and localization signals. Three examples of distinct SLO niches are depicted.

**Figure 4.**
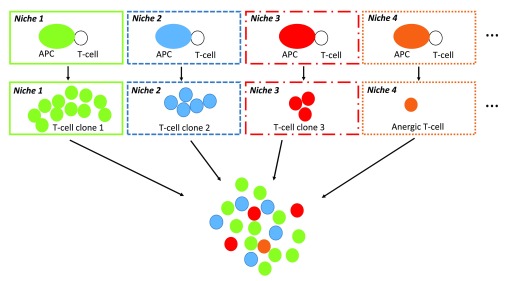
Integrated immune perception at the scale of SLOs. The interaction between APCs and epitope-specific T-cells occur in a multitude of niches that provide distinct combinations of output signals from primary perception tasks. In each niche, these output signals are integrated at the single cell level by epitope-specific T-cells. This step results in the generation of a large array of TH or CD8 T-cell subsets that possibly include anergic T-cells. Eventually, the integrated perception of IOs is performed at the scale of SLOs by a large variety of antigen-specific T-cell and B-cell clones. Only TH-cell clones are depicted in the diagram.

The multitude of niche-specific integration processes that are performed at the single cell level in SLOs is likely to generate a variety of T-cell subpopulations harboring distinct functional behaviors (TH1, TH2, TH17, TFH, Treg…) and recognizing distinct immunodominant epitopes ( Figure 3 and Figure 4). Supporting this view, two recent important studies definitively demonstrated that: i) CD4 T cells primed *in vivo* by pathogens or vaccines are highly heterogeneous with regard to TCRs and TH profiles ^56^, ii) germinal center reactions in response to complex antigens generate a highly diverse B-cell population in terms of BCR affinity ^57^. Thus, at the scale of a SLO the integrated perception of an IO relies on a multitude of antigen-specific T-cell and B-cell clones that provide a whole of "angles of view" from the same immune object. Whether or not such a diversity is progressively narrowed during the re-occurrence of an immune object (i.e during recall immune perception and recall immune response) would require investigations.

**Figure 5.**
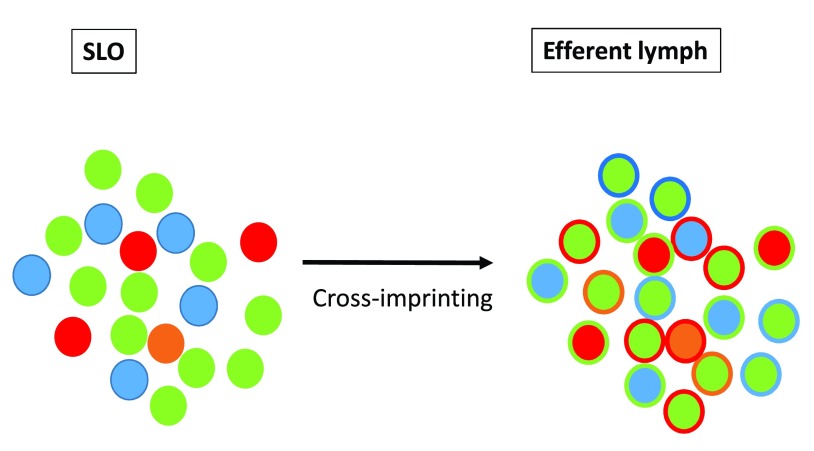
Higher order integrated immune perception. Higher order integration tasks may take place in the efferent lymphatic system. This process is proposed to rely on interclonal communications leading to a functional "cross-imprinting" of TH cells.

It is now recognized that a relatively high level of functional plasticity is maintained in transcriptionally committed TH cell subsets ^58– 62^ as well as in CD8 cytotoxic T-cells ^63^ and B-cells ^64^. While SLOs orchestrate an integrated immune perception of IOs, one may postulate that higher order integration steps may take place in the efferent lymphatic system. This process would rely on interclonal communications leading possibly to a functional "cross-imprinting" of TH cells ( Figure 5).

### The integrated perception of immune objects is performed at the scale of SLOs

At the single cell level, a large number of immune cells may integrate antigenic, temporo-contextual and/or localization signals within or outside SLOs. Besides TH cells and CD8 T-cells, these include B-cells and plasma cells as well as NKT-cells and γδ T-cells. Moreover, recent findings indicate that innate myeloid or lymphoid cells may also integrate and memorize distinct categories of primary immune signals ^65^. However, the immune perception theory sheds a new light on the obvious although frequently neglected statement that SLOs are indispensable to the generation of any integrated immune response and, in the context of perceptive immunity, any integrated immune perception. Indeed SLOs harbor a unique ability to: i) concentrate a large array of cells involved in primary integration tasks, ii) provide a multitude of niches for single cell integration processes.

### Immune perception orientates and adjusts decision making in the immune system

**Figure 6.**
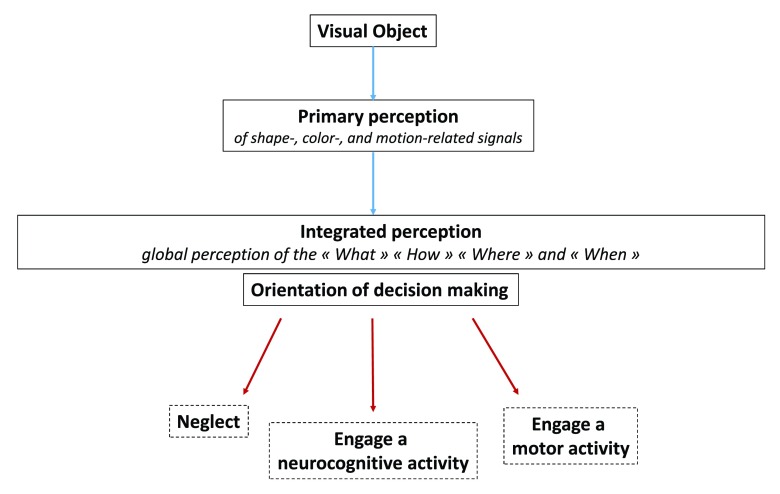
Functional diagram of the visual system. Distinct primary perception tasks allow the shape, color and motion of a visual object to be perceived separately. Output signals generated from this primary perception step instruct an integrated perception allowing the “What”, “How”, “When” and “Where” of a visual object to be perceived as a whole. Such an integrated perception orientates decision making along three main axes: 1) neglect, 2) engage a neurocognitive activity (memorization, attention, thoughts, emotions…) or 3) engage a motor activity (grasp, repel, approach, flee…).

The sensory nervous system allows perceiving as a whole the identity and nature of visual objects, their precise localization, visual context and time-related features (motion, memory traces). Visual perception and other facets of our sensory skills are functionally crucial in the orientation of decision making. Such an orientation may schematically follow three main axes: 1) neglect, 2) engage a neurocognitive activity (memorization, attention, thoughts, emotions…), 3) engage a motor activity (grasp, repel, approach, flee…) ( Figure 6). Of note, visual perception is a dynamic process that not only orientates but continuously adjusts decision making. Thus, motor activity and visual perception are finely coupled via a whole of feedforward and feedback mechanisms allowing the execution of motor programs to be adjusted ^66^. In a similar manner, the somatosensory perception of movements is essential to the control of motor activity ^67^.

**Figure 7.**
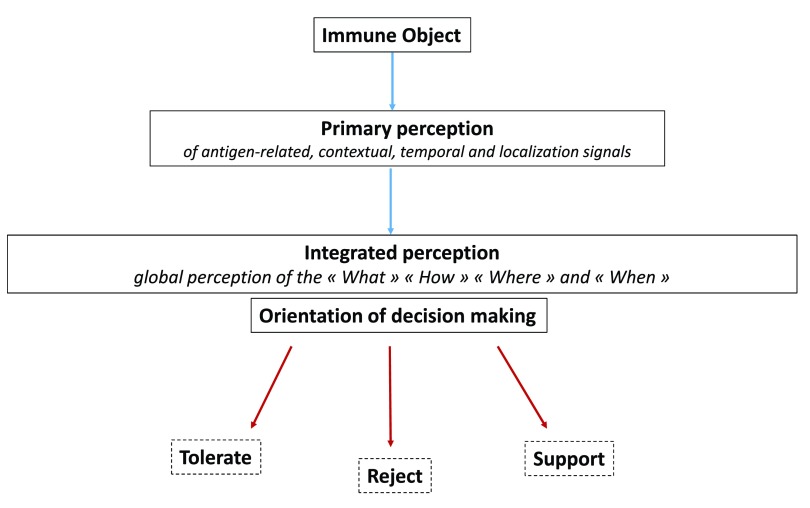
Functional diagram of the sensory immune system. Distinct primary perception tasks allow the antigenic, temporo-contextual and localization signals characterizing an immune object to be perceived separately. Output signals generated from this primary perception step instruct an integrated perception allowing the "What", "How", "When" and "Where" of an immune object to be perceived as a whole. Such an integrated perception orientates decision making along three main axes: 1) tolerate, 2) reject, 3) support. i.e provide molecular and cellular instructing signals that maintain homeostasis or favor tissue repair.

Similar to sensory neural perception, it may be suggested that the main function of immune perception is to orientate decision making toward the engagement of proper immune responses or immune programs. When bringing out the danger theory ^68, 69^, Polly Matzinger was the first to emphasize the importance of contextual inputs in the initiation of "reject" vs "tolerate" immune responses. Since then, the concept of “protective autoimmunity” enunciated by Michal Schwartz and Jonathan Kipnis ^70^ stated that the recognition of tissue-specific auto-antigens allows the immune system to provide a tissue-specific support that is shaped by contextual signals ^71, 72^. A semantic adjustment to these major conceptual advances would consist in proposing that contextual inputs orientate decision making along 3 main axes: "Reject", "Tolerate" or "Support" i.e provide molecular and cellular instructing signals that maintain homeostasis ^73– 75^ or favor tissue repair ^76, 77^. A functional diagram of immune perception and decision making in the immune system could be then aligned with the model of visual perception and decision making in the nervous system ( Figure 7). Along this line, it may be proposed that, similar to the visuomotor and sensorimotor feedback processes, effector immune cells that may be drained from tissues to SLOs deliver output signals reflecting the execution of immune programs. The efferent phase of any immune response would be thus constantly adjusted via feedback signals that are captured and integrated in SLOs.

### Conclusion

The immune perception theory proposes that immunity is driven by several basic principles that are shared between the immune and nervous system. The first proposed principle is that immune cells are not only recognizing antigens +/- danger signals but are indeed perceiving immune objects that are formed by a whole of antigenic, contextual, temporal and localization signals. The second proposed principle is that immune signals are not only individually captured by immune cells but collectively integrated at the scale of SLOs. Such a cooperative functional organization holds relevance for the communications between innate and adaptive immune cells but also for the interactions between T-cell and B-cell clones that recognize a common immune object. The third basic principle is that immune perception is shaped by a number of parameters that are independent from the perceived immune objects ^11^. These include notably the age, gender, metabolic status and gut microbiota composition of the host.

Over the last decades, the research fields covered by immunology have considerably expanded along with the number of breakthrough discoveries relating with the immune system. As a consequence, capturing an up-to-date global image of the immune system functions has become an increasingly difficult task for education professionals and for students as well. In this regard, the theoretical framework proposed here may be essentially considered as a potentially valuable tool for the teaching of immunology. In addition, while neuroimmunology encompasses, for the most part, the study of neuroimmune interactions, the present work suggests that a larger partnership could be envisioned between neuroscientists and immunologists, on the realm of education. To face the challenge of intimately understanding complex systems such as the immune and nervous systems, a move toward an educational approximation of both disciplines is possibly of major importance to promote future cross-fertilizations of ideas and concepts.
