# Supplementary material for: Clonal selection versus clonal cooperation: the integrated perception of immune objects
Source: F1000Res. 2016 Sep 5;5:2226. [Version 1] doi: 10.12688/f1000research.9386.1 (PMC5081160; doi:10.12688/f1000research.9386.1)
Supplement: Peer review history

# Peer review: Referee response for version 1

**DOI:** 10.5256/f1000research.10110.r16681

**Published:** 2016-10-12

**Authors:** Alfred I. Tauber

**Affiliations:** 1 Center for Philosophy and History of Science, Boston University, Boston, MA, USA

Serge Nataf has admirably schematized the “cognitive paradigm,” so aptly coined by Irun Cohen 25(!) years ago (see his citations in the accompanying review). The immune system is, indeed, a “mobile” brain (Fridman 1991) and the parallels between the immune and nervous systems is far from metaphorical or idealized modeling: their inter-connections include the origins of their respective developments, that continue with intimate anatomic relationships, as well as shared messengers and receptors. Indeed, *psychoneuroimmunology* comprises a large research field, whose wealth of studies have confirmed the close evolutionary histories of what are, in essence, similar information processing systems (Ader, Felten and Cohen 1991; Ader 2006; Orosz 2001; Forrest and Hofmeyr 2001). (Note, instead of using the visual system as his example, Nataf might have shown the obvious similarities in the molecular-sensing perception of taste or smell to make the same point about perception and, in that example, emphasized the similarities of structural correspondence and subsequent activation employed by both the immune and taste systems.)

 Two historical notes:

 First, while Nataf credits Jerne with introducing cognitive terminology to immunology (citing the 1974 idiotypic network paper), the origins of immunity as *cognitive* appears earlier in the cybernetics craze of the early 1950s and Burnet’s musings a decade later (Tauber 1994). (Although Burnet first used "cognate" in passing in 1959 [Burnet 1959, p.70], by 1962, he invoked an analogy with language to account for antibody selection [Burnet 1962, p. 94-5].) But historical primacy is not the matter of interest regarding Jerne’s seminal paper, but rather his introduction of how the *context* of antigen presentation determined the immune response. According to his hypothesis, the disruption of the network by antigen, and more to the point, the extent of that interruption in terms of breaking the inter-connections of the lattice-like structure, determined the immunogenicity of the introduced substance. That fundamental idea is the source of Matzinger’s Danger Theory and Pradeu’s more recent continuity/discontinuity theory.

 The second historical note is that an important chapter of the cognitive paradigm’s history must include the work of Antonio Coutinho (1991; 1995; 2003), Francisco Varela (Varela *et al.,* 1988; 1991, 1993; Varela and Coutinho 1991), John Stewart (1992; 1994a; 1994b; Stewart and Varela 1989), and Nelson Vaz (2016; Vaz *et al.,* 2006). These publications developed Jerne’s cognitive point in its full theoretical array and attempted to draw both theoretical and practical parallels between the nervous and immune systems. Those studies comprise the rich mulch for the emergence of our current thinking about how immunity may be properly regarded as an information processing faculty and as such shares organizational (and ultimately regulatory) characteristics with the brain.

 In terms of going beyond Nataf’s schema as an educational tool (which it certainly is), a deeper assessment must be made as to how well modelers have applied the integrative principles employed here. In other words, to the extent systems biology has offered models of immune responses or steady-state conditions, has the hierarchical structure employed by Nataf been utilized and if so, what insights does such an approach offer? That assessment would be useful not only in terms of confirming Nataf’s schema, but perhaps modelers might develop this approach for their own efforts. A review of this issue, as well as a critical discussion about the cognitive paradigm applied to immunology, is found elsewhere (Tauber 1997; 2013; 2017).

I have read this submission. I believe that I have an appropriate level of expertise to confirm that it is of an acceptable scientific standard.

## References

[1] AderR.: Psychoneuroimmunology (4th ed.). *Elsevier Academic Press*.2006; Reference source

[2] AderR.FeltenD.L.CohenN.: Psychoneuroimmunology (2nd ed). *Academic Press*.1991;

[3] CoutinhoA: Size and connectivity: a bit of the history of immune networks. *J Theor Biol*.1991;149(3) :425-7 184289510.1016/s0022-5193(05)80314-5

[4] CoutinhoA: The network theory: 21 years later. *Scand J Immunol*.1995;42(1) :3-8 763114110.1111/j.1365-3083.1995.tb03619.x

[5] CoutinhoA: A walk with Francisco Varela from first- to second-generation networks: in search of the structure, dynamics and metadynamics of an organism-centered immune system. *Biol Res*.2003;36(1) :17-26 1279520510.4067/s0716-97602003000100004

[6] FridmanW.H.: Le Cerveau Mobile. *Paris: Hermann*.1991;

[7] StewartJ.: Immunoglobulins did not arise in evolution to fight infection. *Immunol Today*.1992;13(10) :396-399 Reference source 141837510.1016/0167-5699(92)90088-O

[8] StewartJ.: The Primordial VRM System and the Evolution of Vertebrate Immunity. *R.G.Landes, Austin, Texas*.1994a;124: Reference source

[9] StewardJ.: Cognition without neurones: adaptation, learning and memory in the immune system. *Cognitiva*.1993;5(2) :187-202 Reference source

[10] StewartJVarelaFJ: Exploring the meaning of connectivity in the immune network. *Immunol Rev*.1989;110:37-61 267684810.1111/j.1600-065x.1989.tb00026.x

[11] TauberA.I.: The Immune Self. Theory or metaphor?. *Cambridge University Press*.1994; Reference source

[12] TauberAI: Historical and philosophical perspectives concerning immune cognition. *J Hist Biol*.1997;30(3) :419-40 1161979010.1023/a:1004247922979

[13] TauberAI: Immunology's theories of cognition. *Hist Philos Life Sci*.2013;35(2) :239-64 24466634

[14] TauberA.I.: Immunity: The Evolution of an Idea. *Oxford University Press*.2017;

[15] VarelaFAnderssonADietrichGSundbladAHolmbergDKazatchkineMCoutinhoA: Population dynamics of natural antibodies in normal and autoimmune individuals. *Proc Natl Acad Sci U S A*.1991;88(13) :5917-21 206287010.1073/pnas.88.13.5917PMC51989

[16] VarelaFJCoutinhoA: Second generation immune networks. *Immunol Today*.1991;12(5) : 10.1016/S0167-5699(05)80046-5 159-66 10.1016/S0167-5699(05)80046-5 1878127

[17] VarelaF.CoutinhoA.DupireB.NelsonN.: Cognitive networks: Immune, neural, and otherwise, in A.S. Perelson (ed.). *Theoretical Immunology, Part II. SFI Series on the Science of Complexity. Addison-Wesley*.359-375

[18] CarelaF.CoutinhoA.StewartJ.: What is the immune network for? in W.D. Stein and F.J. Zerla (eds.), Thinking About Biology, Studies in the Science of Complexity, Lecture Notes Vol. III. Redwood City, CA. *Addison-Wesley Publishing Co.*.1993;215-230

[19] VazNM: Self-tolerance revisited. *Stud Hist Philos Biol Biomed Sci*.2016;55:128-32 2720044310.1016/j.shpsc.2015.11.006

[20] VazNMRamosGCPordeusVCarvalhoCR: The conservative physiology of the immune system. A non-metaphoric approach to immunological activity. *Clin Dev Immunol*.13(2-4) : 10.1080/17402520600877216 133-42 10.1080/17402520600877216 17162356PMC2270771

# Peer review: Referee response for version 1

**DOI:** 10.5256/f1000research.10110.r16829

**Published:** 2016-10-06

**Authors:** Irun R. Cohen

**Affiliations:** 1 Department of Immunology, Weizmann Institute of Science, Rehovot, Israel

The paper by Serge Nataf, *Clonal selection versus clonal cooperation: the integrated perception of immune objects*, is aptly named: here Nataf draws an analogy between the immune system (IS) and the central nervous system (CNS); both systems, he points out, are cognitive in the sense that they construct their objects of attention through the integration of discrete features perceived individually. The CNS *constructs* specific visual objects by *integrating* information between networks of neurons that gather discrete and separate information about shape, location, color and motion; similarly, the IS constructs immune objects by integrating discrete information about antigens, cytokines, chemokines, and other signals obtained separately by various cell types such as macrophages, dendritic cells, and B and T lymphocytes. Integration of information between different cells and cell types requires cooperation between the interacting cells; thus the IS, like the CNS, makes functional decisions based on clonal cooperation and not on clonal competition for survival of the fittest. The cognitive analogy is presented in a clear and stimulating manner. However, I think that Serge Nataf could increase the impact of his CNS-IS discussion by sharpening or expanding some additional points:

- *Functional development requires somatic experience*: Both the CNS and the IS combine individual somatic experience with innate programming acquired through evolution of the species. In fact, the CNS and the IS are the only two mammalian systems that require post-natal, individual contact with the world to self-organize their mature structures and functions (see Cohen I.R, (2000)); an IS deprived of immune experience remains undeveloped as does a CNS deprived of formative stimulation.
- *Homunculi direct attention to environmental information that serves adaptive fitness*: Nataf mentions briefly that both the CNS and the IS use homunculi – internal representations of selected features of the self and the outside world; he might add that such homunculi exist to maximize evolutionary fitness. The human brain, for example, is born hard-wired to be attracted to human faces; this primitive homuncular sense of facial recognition is sharpened by individual post-natal experience with real persons, beginning with mother and leading to success in interpersonal bonding, friendship and other important relationships, and to the capacity to communicate by facial expressions.
- The immunological homunculus, which is encoded in innate immune receptors and in shared autoantibody repertoires (Madi A, *et al.* (2015)) and in public T cell receptor repertoires (Madi A, *et al.* (2014)) is much less appreciated. But, like the Neurological Homunculus, these congenital and acquired receptors are likely to enhance fitness, for example, by enabling the IS to better preserve and heal the self and its microbiome while protecting the body from pathogenic invaders from without and from tumor cells from within.
- Important points of difference between the CNS and IS would include hard wiring compared to cell migration, reaction times of milliseconds to hours and days, markedly different cell turnover and differentiation rates, and others; both systems create and integrate their perceived objects in markedly different ways. But, as Nataf points out so convincingly, integrated cooperation is the hallmark of both systems. 

I have read this submission. I believe that I have an appropriate level of expertise to confirm that it is of an acceptable scientific standard.

## References

[1] CohenI.R.: Tending Adam's Garden: evolving the cognitive immune self. *Academic Press*.2000; Reference source

[2] MadiABransburg-ZabarySMaayan-MetzgerADarGBen-JacobECohenIR: Tumor-associated and disease-associated autoantibody repertoires in healthy colostrum and maternal and newborn cord sera. *J Immunol*.2015;194(11) : 10.4049/jimmunol.1402771 5272-81 10.4049/jimmunol.1402771 25917091PMC4432729

[3] MadiAShifrutEReich-ZeligerSGalHBestKNdifonWChainBCohenIRFriedmanN: T-cell receptor repertoires share a restricted set of public and abundant CDR3 sequences that are associated with self-related immunity. *Genome Res*.2014;24(10) : 10.1101/gr.170753.113 1603-12 10.1101/gr.170753.113 25024161PMC4199372
